# Behçet’s Disease, Pathogenesis, Clinical Features, and Treatment Approaches: A Comprehensive Review

**DOI:** 10.3390/medicina60040562

**Published:** 2024-03-29

**Authors:** Salvatore Lavalle, Sebastiano Caruso, Roberta Foti, Caterina Gagliano, Salvatore Cocuzza, Luigi La Via, Federica Maria Parisi, Christian Calvo-Henriquez, Antonino Maniaci

**Affiliations:** 1 Faculty of Medicine and Surgery, University of Enna “Kore”, 94100 Enna, Italy; salvatore.lavalle@unikore.it (S.L.); caterina.gagliano@unikore.it (C.G.); 2 Department of Medical and Surgical Sciences and Advanced Technologies “GF Ingrassia”, ENT Section, University of Catania, Via S. Sofia, 78, 95125 Catania, Italy; sebastiano.caruso2404@gmail.com (S.C.); s.cocuzza@unict.it (S.C.); federicamariaparisi@gmail.com (F.M.P.); 3 Division of Rheumatology, A.O.U. “Policlinico-San Marco”, 95123 Catania, Italy; robertafoti@hotmail.com; 4 Ophthalmology Clinic, San Marco Hospital, University of Catania, 95123 Catania, Italy; 5 Department of Anaesthesia and Intensive Care, University Hospital Policlinico-San Marco, 24046 Catania, Italy; luigilavia7@gmail.com; 6 Service of Otolaryngology, Hospital Complex of Santiago de Compostela, 15701 Santiago de Compostela, Spain; christian.calvo.henriquez@gmail.com

**Keywords:** Behçet’s disease, pathogenesis clinical features, treatment approaches, epidemiology

## Abstract

Behçet’s disease is a systemic inflammatory disorder of unknown etiology. The disease manifests with diverse clinical symptoms, most commonly recurrent oral and genital ulcers, skin lesions, and uveitis, though it can affect multiple organ systems. Diagnosis is primarily clinical due to the lack of a definitive diagnostic test, and management involves a multidisciplinary approach to control inflammation and manage symptoms. Current treatment strategies involve corticosteroids, immunosuppressive agents, and, increasingly, biological therapies. Behçet’s disease exhibits a higher prevalence along the Silk Road, suggesting a role of environmental and genetic factors. Despite significant progress in understanding its clinical characteristics and treatment approaches, gaps remain in our understanding of its pathogenesis. Future research is needed to elucidate the disease’s pathophysiology and optimize treatment strategies.

## 1. Introduction

Behçet’s disease, first identified by the famous Turkish dermatologist Hulusi Behçet [1], is a systemic vasculitis characterized by recurrent oral and genital ulcers, skin lesions, and uveitis [2]. This multisystem disease can affect various organs, including the eyes, skin, joints, gastrointestinal tract, and central nervous system [3]. Although not geographically restricted, the disease is more prevalent in countries along the ancient Silk Road, extending from Eastern Asia to the Mediterranean basin [4]. The pathogenesis of Behçet’s disease remains mysterious [5], believed to result from an abnormal immune response triggered by an environmental agent in a genetically susceptible individual [6]. The disease’s genetic association is highlighted by its prevalence in families with a history of Behçet’s and individuals carrying the HLA-B51 allele, a genetic marker associated with it [7]. Clinically, Behçet’s disease presents a diagnostic challenge due to its diverse range of symptoms, varying significantly among patients [8]. The hallmark feature of Behçet’s disease is painful recurrent oral ulceration, which affects over 95% of patients and often heralds disease onset [3]. Less frequently, genital ulcers arise on the scrotum or vulva, healing with scars. Other mucocutaneous findings include erythema nodosum-like lesions, acneiform nodules, and pathergy reaction. Ocular involvement, which can culminate in blindness, manifests as anterior or posterior uveitis. Musculoskeletal involvement is common, with arthralgia and arthritis usually affecting the knees, ankles, and wrists [4]. Gastrointestinal, vascular, and neurological involvement varies in incidence and severity between ethnic groups [5,9]. The variability in clinical presentation can lead to delayed diagnosis, which can have serious implications given the potential severity of the disease, particularly when it involves the nervous system or eyes [10,11,12]. This remarkable diversity in organ involvement combined with the relapsing–remitting course creates substantial diagnostic challenges. The International Study Group criteria established in 1990 require oral ulcers plus two of four key findings—recurrent genital ulcers, eye lesions, typical skin lesions and pathergy positivity [6] (Table 1).

However, patients often present with atypical features necessitating a high index of clinical suspicion. Early diagnosis is imperative to prevent irreversible end-organ damage, particularly ocular disease which can rapidly lead to blindness [7]. The etiopathogenesis of Behçet’s disease has evaded elucidation despite decades of research. Current evidence implicates environmental triggers eliciting aberrant innate and adaptive immune responses in genetically predisposed individuals [8]. Altered neutrophil and T-cell activation, polarization of pro-inflammatory cytokines, and loss of immune tolerance are believed to drive the inflammatory cascade [9]. The HLA-B51 allele confers the strongest genetic susceptibility, though non-HLA genes are also implicated [10]. Ongoing research seeks to decipher trigger factors, delineate immunological pathways, and identify biomarkers and targets for novel treatments [11]. The management of Behçet’s disease is complex, typically requiring a multidisciplinary approach [13,14,15]. Treatment strategies primarily aim to control inflammation and manage symptoms, with the choice of therapeutic agents often guided by the specific organ systems involved and the severity of the disease [16,17]. Common treatments include corticosteroids, immunosuppressive agents, and newer biological therapies [18]. Despite significant advancements in understanding Behçet’s disease’s clinical features and management strategies, much remains to be discovered about its pathogenesis and optimal treatment approaches [19,20]. While remission is achievable for many with modern management, others experience blindness, vascular events, neurological damage, and gastrointestinal complications [15]. Sustained collaborative efforts integrating clinical acumen and scientific discovery are key to unlocking the remaining mysteries of this fascinating disease at the crossroads of rheumatology, dermatology, and ophthalmology. The advent of precision medicine and systems biology approaches promises new horizons in understanding Behçet’s intricate puzzle to ultimately improve patient outcomes. This comprehensive review explores these aspects by interpreting a reference list. However, due to the lack of raw data and specific content from the original articles, this review might not fully represent the results and conclusions drawn in the initial studies.

## 2. Materials and Methods

This comprehensive literature review was undertaken to collate and elucidate information pertaining to Behçet’s disease, with a particular emphasis on its pathogenesis, clinical manifestations, and therapeutic approaches. In pursuit of pertinent studies, an exhaustive search was executed across different electronic databases, including PubMed, Embase, and Scopus. The search protocol was meticulously crafted to encompass studies released up to 1 February 2024, deliberately eschewing any language constraints. Employed search terms and their permutations included ‘Behçet’s disease’, ‘Behçet’s syndrome’, ‘ocular manifestations’, ‘mucocutaneous manifestations’, ‘vascular manifestations’, ‘central nervous system involvement’, ‘diagnosis,’ ‘treatment’, and ‘clinical trials’. In addition, the bibliographies of pertinent articles and reviews were scrupulously examined to uncover any studies that might have escaped initial detection. The data interpretation was predicated upon an extensive analysis of the accessible information from each cited source. Thus, the insights gleaned from the review were synthesized, and a series of probing questions were articulated to thoroughly evaluate each principal aspect of Behçet’s disease: (1) What is the typical presentation of Behçet’s disease in adults? (2) What causes Behçet’s disease? (3) What diagnostic tests are recommended for Behçet’s disease? (4) How is Behçet’s disease managed and treated? (5) What is the prognosis of Behçet’s disease? (6) What are the potential complications of Behçet’s disease? (7) What lifestyle modifications can help manage Behçet’s disease? (8) What is the epidemiology of Behçet’s disease? (9) What is the impact of Behçet’s disease on mental health? (10) Can Behçet’s disease affect pregnancy and fertility?

The purpose of this review was to provide detailed and easily accessible information through the interpretation of available information in the literature on Behçet’s disease. 

## 3. Results

### 3.1. What Is the Typical Presentation of Behçet’s Disease in Adults?

**Figure 1:**
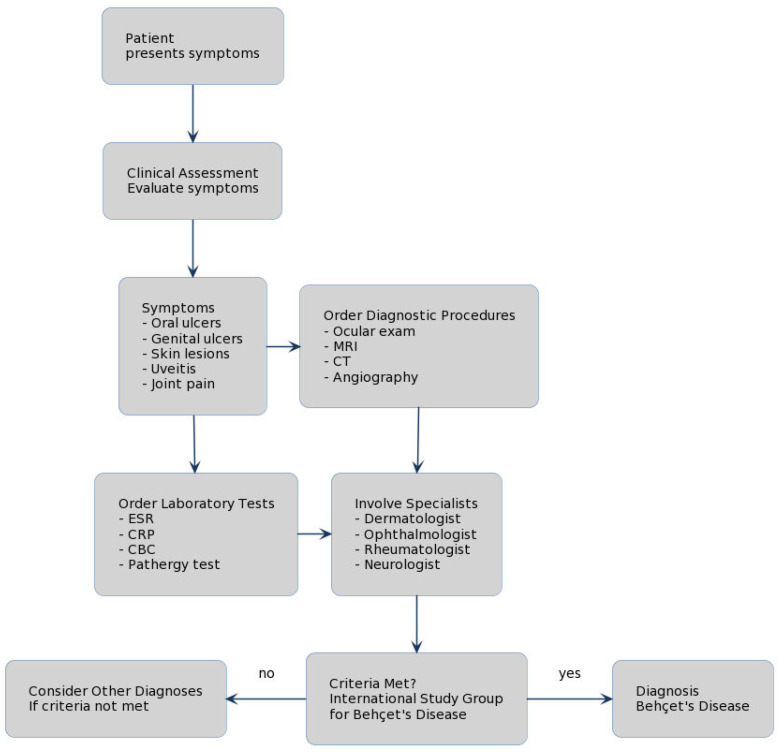
Flow-diagram. Behçet’s diagnosis of clinical symptoms, oral, skin, and genital signs. A multidisciplinary approach is mandatory.

Behçet’s disease is a complex disorder that affects various systems in the body, with varying clinical features from patient to patient [21]. The formation of these ulcers is thought to be due to a dysregulation of the immune system, leading to increased production of inflammatory cytokines such as TNF-alpha and IL-6, which damage the mucosal lining [22]. Almost all patients experience recurrent painful oral aphthous ulcers, typically the first symptom of the disease (Figure 1).

Less frequently, patients may also experience painful genital ulcers, which may scar after healing. These genital ulcers are believed to be caused by similar immunological disturbances as oral ulcers, involving a T-cell-mediated immune response [23]. Another common feature is ocular involvement, specifically anterior and posterior uveitis. The pathogenesis of uveitis in Behçet’s disease is linked to an autoimmune response, where immune complexes deposit in the uveal tract and trigger an inflammatory cascade, potentially involving molecular mimicry with heat-shock proteins [24]. This can present as redness, pain, and blurred vision, and if not treated, can lead to blindness. Additionally, skin lesions such as erythema nodosum-like lesions, acneiform nodules, and pseudofolliculitis can occur [25]. These lesions are associated with vasculitis, and the inflammation is often driven by neutrophilic infiltration and the release of proteolytic enzymes, further contributing to the skin manifestations [26]. Vascular involvement can include venous and arterial thrombosis, aneurysms, and varices. The vascular pathology is characterized by endothelial dysfunction, increased endothelial expression of adhesion molecules, and an imbalance in procoagulant and anticoagulant factors leading to a hypercoagulable state [27]. Joint involvement, particularly arthralgia or arthritis of the knees and ankles, is common [28]. This may be due to immune complex deposition in the synovium or direct attack by autoreactive T-cells, causing synovitis and articular inflammation [29]. Gastrointestinal symptoms such as abdominal pain, diarrhea, and gastrointestinal bleeding due to intestinal ulcers can also occur [30]. The ulcers are often a result of vasculitis in the small or large intestine, leading to tissue necrosis and ulceration [31]. Neurological manifestations can include headaches, strokes, meningitis, or encephalitis [32,33,34]. These can be attributed to vasculitis affecting the central nervous system, with inflammation and possible thrombosis disrupting normal neurological function [35].

### 3.2. What Is the Epidemiology of Behçet’s Disease?

Behçet’s disease is relatively rare and has a distinct geographical distribution. It is most prevalent in countries along the Silk Road, which stretches from East Asia to the Mediterranean basin. This includes countries like Turkey, Iran, Japan, and Korea [36]. The disease affects both men and women, although the disease course may be more severe in men. Typically, symptoms begin when individuals are in their 20s or 30s [37]. However, the disease can start at any age. The prevalence and incidence rates vary greatly between regions, with Turkey having the highest rates [38].

### 3.3. What Causes Behçet’s Disease?

The exact cause of Behçet’s disease is not clearly understood. Still, it is believed to be due to a combination of genetic and environmental factors due to the higher prevalence of the disease in countries along the ancient “Silk Road” from the Middle East to East Asia [39]. Numerous genetic loci have been linked by genome-wide association studies to an elevated chance of developing Behçet’s illness; particularly the HLA-B51 allele, is associated with an increased risk of developing Behçet’s disease [40]. Compared to approximately 10–20% of healthy controls, up to 60% of individuals with Behçet’s disease carry the HLA-B51 allele. Behçet’s illness has also been connected to other genetic variations, including those in the IL-10, IL-23R, and ERAP1 genes [41,42,43,44,45]. However, the disease cannot be caused just by these genetic markers, indicating that environmental variables are also important in the development of disease. Behçet’s disease may be triggered by infectious pathogens in the environment, specifically Streptococcus sanguis and herpes simplex virus [44]. In people who are genetically predisposed to the disease, these infections may trigger an aberrant immune response. Behçet’s illness may also develop as a result of other environmental variables, including smoking, stress, and food [24,26,46]. Innate and adaptive immune responses are involved in immunological abnormalities, which are at the core of Behçet’s disease pathogenesis [47]. It has been demonstrated that innate immune cells, such as neutrophils and monocytes, activate more quickly and produce more pro-inflammatory cytokines, such as IL-1β, IL-6, and TNF-α [41]. These cytokines are involved in the long-term inflammatory condition seen in Behçet’s illness. Patients with Behçet’s disease have been shown to have neutrophil hyperactivity, which is characterized by enhanced chemotaxis, phagocytosis, and superoxide generation [20]. Furthermore, it has been demonstrated that, in comparison to healthy controls, the monocytes from Behçet’s disease patients produce higher levels of IL-1β, IL-6, and TNF-α [6]. A key player in regulating adaptive immunological responses, T helper (Th) cells have been linked to the etiology of Behçet’s illness [23]. Behçet’s illness was once thought to be a Th1-mediated inflammatory condition marked by elevated IFN-γ production. However, according to new research, Th17 cells—which also secrete IL-17 and IL-22—may be crucial in the illness [22,42,43]. Specialized antigen-presenting cells are assumed to be the driving force behind the formation of Th17 cells, which have been demonstrated to be pathogenic in numerous autoimmune and inflammatory disorders in humans [41]. Elevated levels of IL-17 and IL-22 have been found in the serum and impacted tissues, including skin lesions and mouth ulcers, in Behçet’s disease [44]. A key role in the pathophysiology of Behçet’s disease appears to be played by the interplay between the innate and adaptive immune systems. For instance, it has been demonstrated that innate immune cells’ production of IL-1β and IL-23 stimulates Th17 cells’ development and growth [43]. The interplay between innate immunity and adaptive immunity could sustain the long-term inflammatory condition seen in Behçet’s. Behçet’s disease has also been linked to regulatory T cell (Treg) dysfunction, which is implicated in immunological homeostasis maintenance [33]. The uncontrolled inflammation and autoimmunity seen in the condition may be attributed in part to impaired Treg activity.

### 3.4. What Diagnostic Tests Are Recommended for Behçet’s Disease?

Diagnosing Behçet’s disease can be challenging due to its diverse clinical manifestations and the absence of a definitive diagnostic test. The diagnosis is primarily made based on clinical criteria [13]. The International Study Group criteria for Behçet’s disease require the presence of recurrent oral ulceration plus at least two of the following: recurrent genital ulcers, eye lesions (uveitis or retinal vasculitis), skin lesions, or a positive pathergy test [42]. A pathergy test is a skin hypersensitivity test where a small skin prick is made, and a positive result is the formation of a small red bump or pustule 24–48 h after the prick. Imaging and laboratory tests may be used to assess organ involvement, such as brain MRI for neurological involvement, or to exclude other diseases with similar symptoms. However, there are no specific laboratory abnormalities associated with Behçet’s disease. While certain genes are associated with an increased risk of Behçet’s disease, genetic testing is generally not used for diagnosis. HLA-B51 can support a diagnosis but is not required [43].

### 3.5. How Is Behçet’s Disease Managed and Treated?

**Figure 2:**
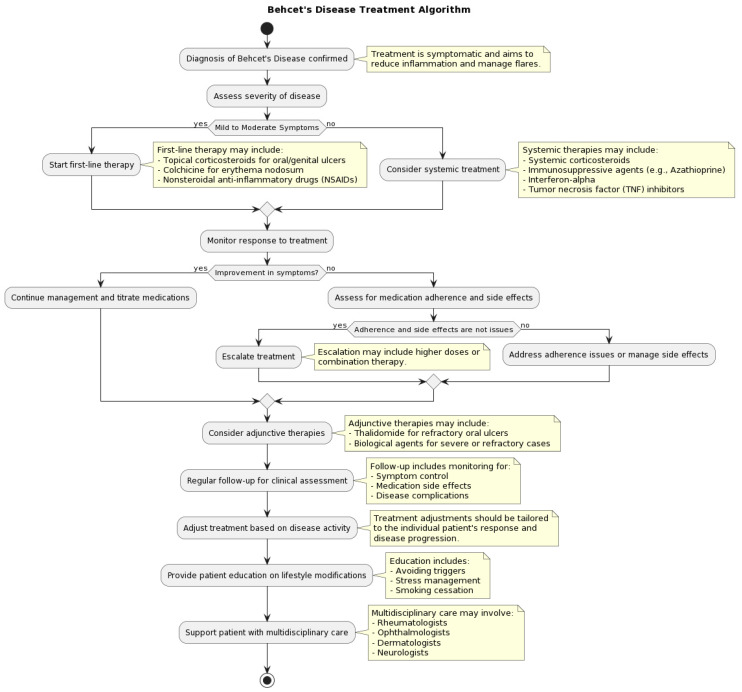
Flow diagram on therapeutic management.

Management and treatment of Behçet’s disease aim to reduce symptoms, prevent flare-ups, and manage complications [44]. Since it is a multisystem disorder, the treatment approach is often individualized depending on the organs involved. Reducing symptoms, averting flare-ups, and managing consequences are the goals of Behçet’s disease management and treatment [44] (Figure 2).

Pain and inflammation may be reduced by nonsteroidal anti-inflammatory medications (NSAIDs) [45]. Depending on the degree and location of symptoms, corticosteroids are frequently used to treat inflammation and can be administered topically, orally, or intravenously [8]. Immunosuppressive medications are frequently used to treat Behçet’s disease, especially in cases that are severe or resistant to treatment [11]. The clinical signs and organ involvement determine which immunosuppressive medication is best [12]. The purine synthesis inhibitor azathioprine has demonstrated efficacy in managing Behçet’s disease symptoms related to the eyes, joints, and mucosa [14]. When compared to a placebo, azathioprine dramatically decreased the incidence and severity of oral and vaginal ulcers as well as arthritis in a randomized controlled trial [48]. The calcineurin inhibitor cyclosporine has been used to treat Behçet’s disease’s ocular symptoms. Cyclosporine successfully decreased the frequency and intensity of ocular episodes in patients with Behçet’s illness, according to research by Masuda et al. [49]. On the other hand, because cyclosporine may have adverse effects such as hypertension and nephrotoxicity, it is necessary to closely monitor its use [50]. The antimetabolite methotrexate has been used to treat Behçet’s disease’s joint and mucocutaneous symptoms. Methotrexate dramatically decreased the frequency and severity of vaginal and oral ulcers as well as arthritis in individuals with Behçet’s illness, according to research by Davatchi et al. [51]. Treatment for the neurological symptoms of Behçet’s illness with methotrexate has also been demonstrated to be successful [52]. Biological therapies that target particular immune pathways have become a promising treatment option for Behçet’s illness in recent times. TNF inhibitors, namely infliximab and adalimumab, have demonstrated efficacy in managing several presentations of Behçet’s disease, such as ocular, joint, and mucocutaneous involvement [47,48]. Infliximab dramatically decreased the frequency and severity of vaginal and oral ulcers as well as arthritis in patients with Behçet’s disease, according to research by Melikoglu et al. [53]. Adalimumab has also demonstrated efficacy in the treatment of Behçet’s disease’s refractory uveitis [54]. Behçet’s disease has also been treated with interferon-alpha (IFN-α), especially in severe or refractory patients. IFN-α significantly decreased the frequency and intensity of ocular episodes in patients with Behçet’s illness, according to research by Kotter et al. [55]. Additionally, it has been demonstrated that IFN-α is useful in treating the neurological and vascular manifestations of Behçet’s illness [56]. Regular follow-ups are essential to track the course of the disease and the effectiveness of treatment, in addition to pharmacological interventions. For the best treatment of Behçet’s illness, rheumatologists, dermatologists, ophthalmologists, and other specialists must work together in multidisciplinary care.

### 3.6. What Is the Prognosis of Behçet’s Disease?

The prognosis of Behçet’s disease varies widely and depends on the severity and location of the symptoms. While it is a chronic condition with periods of remission and flare-ups, most people with Behçet’s disease can lead normal lives with appropriate treatment [28]. The disease tends to be more severe in the early years and may decrease in severity over time. Complications such as blindness and neurological damage can affect the quality of life and prognosis. Death is rare and is usually due to major vessel disease or neurological involvement [45]. Regular follow-up care and monitoring can help manage the condition and prevent complications.

### 3.7. What Are the Potential Complications of Behçet’s Disease?

The range of consequences associated with Behçet’s illness varies based on the body part affected. Patients experience these complications at different rates and in different degrees, and some factors have been linked to a more severe course of the disease. If treatment for eye involvement is delayed, visual loss may result, especially in cases of posterior uveitis and retinal vasculitis. According to a research study by Kaçmaz et al., 50–70% of cases of Behçet’s illness involved the eyes, with bilateral panuveitis being the most typical symptom [57]. Male sex, younger age at initiation, and HLA-B51 positive are risk factors for significant ocular involvement [58]. Corticosteroids, immunosuppressive medications (such as azathioprine, cyclosporine), and biological treatments (such as interferon-alpha, TNF inhibitors) are available for the treatment of ocular problems [59]. Headaches, strokes, and other neurological abnormalities can result from neurological involvement, also referred to as neuro-Behçet’s [58,59]. It is expected that 5–10% of patients with Behçet’s disease also have neuro-Behçet’s [60]. Male sex, younger age at onset, and HLA-B51 positive are risk factors for neuro-Behçet’s [61]. High-dose corticosteroids, immunosuppressive medications (like cyclophosphamide), and biological therapy (like TNF inhibitors) are among the available treatments for neuro-Behçet’s disease [62]. Blood clots, aneurysms, or involvement of the pulmonary artery can arise from vascular involvement [35]. Behçet’s disease has a 7–38% frequency of vascular involvement [63]. Male sex and younger age at initiation are risk factors for vascular problems [64]. Anticoagulation therapy and immunosuppressive medications, such as cyclophosphamide, are available for the treatment of vascular problems. Abdominal pain, bleeding, and perforation can result from gastrointestinal involvement [60]. Behçet’s disease is thought to affect the gastrointestinal tract 3–16% of the time [65]. Male sex and younger age at onset are risk factors for gastrointestinal problems [66]. Corticosteroids, immunosuppressive medications (such as azathioprine), and biological treatments (such as TNF inhibitors) are among the treatment choices for gastrointestinal problems. Scarring may result from severe skin involvement [18]. Up to 80% of individuals with Behçet’s illness will experience cutaneous signs, indicating a high incidence of skin involvement [67]. Male sex and HLA-B51 positive are risk factors for significant skin involvement [68]. Topical corticosteroids, colchicine, and immunosuppressive medications (such as azathioprine) are among the treatment choices for skin problems [69,70,71,72].

### 3.8. What Lifestyle Modifications Can Help Manage Behçet’s Disease?

Several lifestyle modifications can help manage Behçet’s disease and improve quality of life. Regular exercise can help maintain joint flexibility and reduce inflammation [44]. A balanced diet with plenty of fruits and vegetables can support overall health and immune function [73]. Adequate rest is essential, as fatigue is a common symptom. Stress management techniques such as mindfulness, meditation, and yoga can help manage symptoms and reduce the likelihood of flare-ups [74]. Avoiding triggers, whenever known, can also help prevent flare-ups. Regular dental hygiene can help avoid oral ulcers, and protection from sun and heat might help prevent skin lesions [75,76]. Regular follow-up with healthcare providers ensures appropriate management and prompt treatment of flare-ups [77,78,79,80].

### 3.9. What Is the Impact of Behçet’s Disease on Mental Health?

Like many chronic illnesses, Behçet’s disease can have significant psychological impacts. The unpredictability of flare-ups can cause stress and anxiety, as patients may worry about when and how severely they will be affected. The presence of painful symptoms and the potential for serious complications, such as vision loss, can also contribute to anxiety and distress [81]. Moreover, the chronic pain associated with Behçet’s disease can have a profound impact on quality of life and may lead to depression [82]. Ongoing management of Behçet’s disease should, therefore, include psychological support, with interventions such as cognitive-behavioral therapy, support groups, and potentially medication for managing symptoms of anxiety and depression [83].

### 3.10. Can Behçet’s Disease Affect Pregnancy and Fertility?

Fertility in men and women does not seem to be considerably impacted by Behçet’s disease. Nonetheless, the illness may cause difficulties with pregnancy and childbirth. While some women may have fewer or milder flare-ups during pregnancy, others may see an improvement in their symptoms. According to a study by Uzun et al., 40% of women with Behçet’s illness had a decrease in disease activity during pregnancy, whereas 27% of them reported a relapse [84]. Behçet’s illness pregnant women are more likely to experience specific problems. Pregnant women with Behçet’s illness had higher odds of miscarriage (odds ratio (OR) 2.4), cesarean section (OR 1.8), and preterm delivery (OR 2.2) than pregnant women in good health, according to a meta-analysis by Noel et al. [85]. Furthermore, venous thromboembolism during pregnancy is more common in women with Behçet’s disease, with an estimated prevalence of 5–12% [86]. For women with Behçet’s disease, medication management is essential during pregnancy since some of the medications used to treat the condition have side effects that should be avoided for the growing fetus [87]. Pregnancy and lactation are safe times to use colchicine, a medicine used frequently for mucocutaneous symptoms [88]. Other immunosuppressive medications, such cyclophosphamide and methotrexate, on the other hand, are teratogenic and must be stopped before to conception [89]. Although TNF inhibitors like infliximab and adalimumab are thought to pose little danger during pregnancy, each situation should be assessed individually before using them [90]. There are additional hazards associated with vascular involvement, especially deep vein thrombosis, when pregnant [91]. Pregnant and postpartum women with a history of vascular involvement should be thoroughly watched, and they may need to take anticoagulant medication [92,93]. Consequently, if a woman with Behçet’s disease wants to get pregnant, she should do so under the supervision of a medical professional skilled in handling high-risk pregnancies [94]. To maximize illness control, modify medication, and evaluate the risk of complications, preconception counseling is crucial [95]. For Behçet’s disease patients to have a successful pregnancy, rheumatologists, obstetricians, and other experts must work closely together. Despite these challenges, many women with Behçet’s disease have successful pregnancies. A study by Iskender et al. found that 79% of pregnancies in women with Behçet’s disease resulted in live births, with a mean gestational age of 37.5 weeks [94]. However, the study also highlighted the importance of close monitoring, as 33% of the pregnancies required hospitalization due to disease exacerbation or pregnancy complications.

### 3.11. While a Multidisciplinary Approach Is Required for Behçet’s Disease?

The management of Behçet’s disease is challenging for practitioners with its wide-ranging effects on the body and the lack of definitive diagnostic tests and needs different medical professionals [15,17]. The critical role of ophthalmologists cannot be overstated, as they are at the forefront of preventing and treating eye conditions like uveitis that can severely affect vision [74]. Dermatologists, on the other hand, are tasked with the careful handling of the skin and mucosal issues that are so characteristic of this disease [19]. When patients face the threat of vascular issues such as blood clots or aneurysms, the expertise of vascular surgeons and hematologists becomes central. Gastroenterologists step in to tackle the often debilitating gastrointestinal symptoms, varying from discomfort to critical bleeding events [30]. Neurological symptoms, with their vast range of presentations from mild to debilitating, are deftly managed by neurologists. Rheumatologists are often seen as the captains of this ship, particularly when it comes to joint-related symptoms and the broader application of systemic treatments [77,85]. The chronic and potentially disabling nature of Behçet’s disease also brings pain specialists and mental health professionals to the fore, providing much-needed pain and psychological support [73]. Nurses, physical therapists, and patient educators are integral members of the care team, also in keeping a watchful eye on the patient’s overall status, including any side effects from the medication [76].

## 4. Discussion

Behçet’s disease epitomizes medical complexity with its kaleidoscopic clinical manifestations, geographic predilection, and elusive pathogenesis. This multifaceted systemic vasculitis warrants an integrated discussion synthesizing insights on its protean features, diagnostic challenges, evolving treatment landscape, and psychosocial burden [83]. The typical presentation of Behçet’s disease includes recurrent oral and genital ulcers, skin lesions, ocular inflammation, and varied systemic features [96]. Oral ulcers, often the initial manifestation, are painful recurrent aphthous ulcers occurring in over 95% of patients [1]. Genital ulcers, less frequent than oral ulcers, arise on the scrotum or vulva and heal with scarring [2]. Ocular involvement, which can lead to blindness if untreated, presents as anterior or posterior uveitis with redness, pain, and vision loss [3]. Erythema nodosum-like skin lesions, acneiform nodules, and pseudofolliculitis are common cutaneous markers. Joint, vascular, gastrointestinal, and neurological involvement can also occur [4]. While most patients exhibit a combination of these symptoms, tremendous diversity exists in the extent and severity of organ involvement [97]. This heterogeneous presentation often confounds prompt diagnosis. International Study Group criteria require oral ulcers plus two of four hallmarks—genital ulcers, eye lesions, skin lesions, or a positive pathergy test [5]. Pathergy refers to skin hypersensitivity, where a minor trauma like a needle prick elicits a papule or pustule. HLA-B51 positivity supports the diagnosis but is not mandatory [6]. Atypical presentations without overt oral ulcers may necessitate more extensive workup. Diagnostic delay can enable progression, underscoring the need for clinical acumen. The etiopathogenesis of Behçet’s disease remains an intriguing conundrum. Current models implicate environmental triggers in genetically predisposed individuals evoking aberrant innate and adaptive immune responses. The prominent geographic corridor spanning East Asia to the Mediterranean basin points to microbial agents as potential triggers [7]. The higher prevalence in family members of affected individuals and association with HLA-B51 highlight genetic susceptibility [8]. Genome-wide studies have identified variants in IL10, IL23R, CCR1, STAT4, KLRC4, ERAP1, and FUT2 genes as disease risk factors [9].

Recent evidence suggests that Bechet’s disease may be better understood as an autoinflammatory disorder [98,99,100,101]. In 2018, Yazici et al. offered a modern interpretation of Bechet’s syndrome, talking about how it is categorized as an autoinflammatory illness [99]. The pathophysiology of Bechet’s illness is linked to deregulation of the innate immune system, and the authors emphasized the efficacy of treatments that target these pathways, such as IL-1 inhibitors [98,102]. Several studies have highlighted the role of innate immune system dysregulation in the pathogenesis of Bechet’s disease. For instance, genetic studies have identified associations between Bechet’s disease and mutations in genes involved in the regulation of innate immunity, such as MEFV, IL-1β, and IL-10 [100,101,102]. Aktas Cetin et al. looked at the possibility of Th22 and Th17 cells that secrete IL-22 and IFN-γ in Bechet’s illness [103]. According to the study, patients with Bechet’s disease had higher concentrations of these cell types, which may indicate that these cells are involved in inflammation and the intricate interaction between innate and adaptive immune responses. Additionally, patients with Bechet’s disease have been found to have elevated levels of pro-inflammatory cytokines, including IL-1β, IL-6, and TNF-α, which are key mediators of the innate immune response. In particular, IL-1 has been studied as potential treatment target in Behçet’s disease. Anakinra, an IL-1 receptor antagonist, was used to treat a case series of individuals with drug-resistant Bechet’s disease, according to Cantarini et al. [104]. The results of the trial supported the pathophysiological function of IL-1 in Bechet’s disease and the therapeutic potential of targeting innate immunity pathways by demonstrating the effectiveness of anakinra in treating the condition’s symptoms. In addition, a multicenter retrospective study assessing the safety and effectiveness of anti-interleukin-1 therapy in Bechet’s disease was carried out by Emmi et al. [105]. The study demonstrated the efficacy and safety profile of anti-IL-1 therapy in treating different Bechet’s disease manifestations. These findings lend support to the diagnosis of Bechet’s disease as an autoinflammatory disorder and the possibility of treating it by focusing on innate immune pathways. Several lines of emerging evidence indicate that dysregulated innate and T-cell-mediated immune pathways underpin Behçe’s pathogenesis. Neutrophil and monocyte hyperactivity, exaggerated inflammatory cytokine responses, and Th1 skewing have been consistently demonstrated [10]. More recently, Th17 cells were shown to be pathologically upregulated in active Behçe’’s disease [11]. Ongoing research is focused on elucidating triggers of immunopathology, identifying reliable biomarkers, and developing targeted immunomodulatory therapies [12]. Given the systemic nature of Behçet’s disease, the treatment approach is tailored to the specific organs affected and guided by disease severity [106]. Mild presentations may be managed with topical measures and nonsteroidal anti-inflammatory drugs. Low-dose oral corticosteroids are first-line for more extensive disease, with dose escalation for refractory manifestations [13]. Immunosuppressive agents like azathioprine, cyclosporine, cyclophosphamide, or methotrexate are reserved for organ-threatening presentations [14]. In recent years, biological therapies that specifically target inflammatory mediators have emerged as effective options for severe Behçet’s disease. Tumor necrosis factor alpha (TNF-α) inhibitors like infliximab, adalimumab, and golimumab have demonstrated efficacy in ocular, mucocutaneous, joint, vascular, and neurological involvement [15]. Interleukin-1 blocking agents like anakinra and canakinumab have shown promise for vascular and rheumatological manifestations [16]. Rituximab, an anti-CD20 monoclonal antibody has been beneficial in ocular and central nervous system disease [17]. Ongoing trials are assessing newer biologics like interleukin-6 and interleukin-17 inhibitors [18]. Multidisciplinary management, treatment of comorbidities, regular follow-up, and patient education are imperative for optimal outcomes. Lifestyle measures like smoking cessation and psychological support also promote wellbeing. Developing targeted immunotherapies based on disease endotypes and biomarkers is an active research frontier [19]. Sustained remission is achievable with modern management in a majority of patients. However, a subset experiences devastating complications like blindness, vascular events, gastrointestinal perforation, or neurological sequelae. Behçet’s disease displays unique geoepidemiological patterns, with the highest prevalence along the ancient Silk Road between Eastern Mediterranean countries and East Asia. Turkey has the highest prevalence of 420 per 100,000 population [20]. Male sex and young age of onset between 25–35 years is characteristic, although pediatric onset can occasionally occur [21]. Considerable geographical variability exists in incidence and prevalence rates. As with many chronic illnesses, Behçet’s disease exerts significant psychosocial impact. Recurrent painful ulcers, visual loss, and the unpredictability of flares contribute to anxiety and depression in patients [25]. Stigmatization from visible ulcers also takes a toll. Furthermore, medications like corticosteroids evoke side effects that impair quality of life. Multifaceted interventions encompassing psychological counseling, support groups, lifestyle education, and holistic care promote patients’ overall wellbeing [28]. Fortunately, fertility is generally preserved in Behçet’s disease. Both men and women typically have normal reproductive capacity [39]. However, vascular events like deep vein thrombosis are more frequent during pregnancy and require close monitoring. Certain medications need to be discontinued or substituted to minimize fetal risks [40]. While most women have successful pregnancies, a multidisciplinary approach optimizes maternal and neonatal outcomes. Despite progress, significant knowledge gaps persist regarding Behçet’s pathogenesis, biomarkers, treatment, and prognosis. Robust models explaining gene–environment interactions, identification of disease initiating events, and deciphering immunological cascades underlying clinical heterogeneity remain elusive goals [41]. Development of clinically useful biomarkers for diagnostics, disease monitoring, and predicting treatment responses is still in evolution [42]. Therapeutics tailored to molecular subtypes and advanced delivery systems warrant further research [43]. Prognostication also remains imprecise, especially for serious manifestations like vasculo-Behçet’s. Global collaborative networks focused on unraveling Behçet’s mysteries promise to accelerate the pace of discovery and innovations in patient care [44]. Behçet’s disease can lead to diverse complications affecting vision, blood vessels, brain, joints, skin, and gut. A knowledgeable multidisciplinary team optimally prevents and manages these complications through careful follow-up, patient education, early intervention, and holistic care. Ocular involvement in Behçet’s disease can lead to potentially devastating consequences like irreversible blindness. Anterior uveitis presents with red, painful eyes and blurred vision. Posterior involvement causes retinal vasculitis and optic neuropathy [1]. Recurrent episodes promote structural damage, resulting in permanent visual impairment or blindness if not treated aggressively. Cataracts, secondary glaucoma, and macular edema further impair vision. Close ophthalmological follow-up and immediate treatment of inflammatory flares with corticosteroids can mitigate ocular morbidity [2]. Immunosuppressants and biologics help control refractory disease. Vascular inflammation in Behçet’s confers substantial morbidity and mortality risk from events like deep vein thrombosis, aneurysmal dilatation, and arterial occlusions [3]. Venous thrombosis frequently manifests in unusual sites like the vena cava or cerebral sinuses rather than the lower limbs. Arterial disease often affects the pulmonary vasculature but can also involve coronaries, aorta, and peripheral vessels. Aneurysm formation, particularly in the pulmonary arteries, carries high risk of rupture and death [4]. Prompt vascular imaging and aggressive immunosuppression is warranted at the earliest signs of vascular involvement. Anticoagulants, steroids, and biologics help minimize complications [5]. Neurological involvement, termed neuro-Behçet’s disease, can be one of the most devastating manifestations. Chronic meningoencephalitis causes cognitive decline and behavioral changes. Brainstem lesions lead to symptoms like dysarthria, hemiparesis, and cranial neuropathy [6]. Spinal cord disease results in weakness and sensory deficits. Optic neuropathy and stroke can arise from vasculitis. Seizures, headaches, and psychiatric disturbances are other neurologic sequelae. Early immunotherapy and careful follow-up aim to limit accrual of neurological deficits [7]. Arthralgia and arthritis, especially of the knees, ankles, and wrists, frequently complicate Behçet’s [8]. Inflammatory joint disease impairs mobility and reduces functional capacity over time. Skeletal complications include osteonecrosis and pathologic fractures. Soft tissue inflammation causes enthesitis and tendonitis. Regular physiotherapy and disease-modifying antirheumatic drugs reduce musculoskeletal morbidity [9]. Recurrent oral and genital ulcers in Behçet’s frequently scar with postinflammatory pigmentary changes. Skin lesions like erythema nodosum and acneiform nodules also heal with dyspigmentation and scarring [10]. Intestinal involvement causes abdominal pain, bloody diarrhea, and ulcers, which can perforate and form fistulas. These complications are challenging to treat and require specialist gastroenterological care [11]. The psychosocial burden of chronic illness is especially relevant for Behçet’s patients. Coping with painful symptoms, uncertain prognosis, and treatment side effects takes a psychological toll. Resultant anxiety, depression, and social isolation are common, but often under-recognized [12]. A supportive approach, with counseling, stress reduction techniques, and peer support, is beneficial alongside medical management. Improving awareness about psychneuroimmunology and biopsychosocial perspectives in Behçet’s care is an evolving need.

## 5. Conclusions/Future Directions

Though significant strides have illuminated many aspects of Behçet’s disease, substantial gaps persist in comprehensively understanding its enigmatic etiopathogenesis and optimizing patient outcomes. Elucidating the inciting factors and intricate immunological pathways underlying Behçet’s skewed geographic distribution and varied clinical phenotypes remains an unfinished goal needing coordinated efforts. Collaborative studies across international cohorts combining genetic, microbiomic, and environmental data could unravel these complex gene–environment interactions. Systems biology approaches integrating multi-omics data with computational models may further disentangle the dynamics of innate and adaptive immune disturbances driving Behçet’s clinical heterogeneity. Another research priority is developing clinically useful biomarkers for early diagnosis, predicting disease course, and selecting targeted therapies. Emerging options like serum microRNAs, metabolomics profiles, and advanced imaging technologies are promising, but warrant validation in diverse ethnic populations. Improved biomarkers could enable precision treatment tailored to disease endotypes and reduce delays in initiating appropriate therapy. Optimizing therapeutic strategies to balance efficacy, safety, and quality of life is an ongoing need. Larger multicenter trials of novel biologics and small molecules are essential to expand the armamentarium against refractory disease. Comparative effectiveness studies of existing immunomodulators could offer evidence-based algorithms guiding therapeutic choices. Evaluating combination and sequential biological treatment also deserves investigation. Finally, research focused on improving understanding of long-term outcomes, complications, and their prevention is imperative. Studies defining the risk factors for blindness, neurological sequelae, vascular events, and other morbidities could improve prognostication and surveillance. Evaluating multimodal interventions incorporating patient education, lifestyle optimization and holistic care alongside medications is vital to reduce disease burden. Realizing the goal of curative solutions for Behçet’s disease requires extensive research on multiple fronts from basic science discovery to clinical therapeutics and beyond. Sustained collaborative efforts powered by creativity, technological innovations, and patient-centric perspectives will illuminate the remaining mysteries around this fascinating disease at the crossroads of medical disciplines.

## Figures and Tables

**Table 1:** The ISG criteria are described. One major symptom plus two of the following should be considered.

Criteria	Description
Recurrent Oral Ulceration	Minor, major, or herpetiform ulceration observed by a physician or patient at least 3 times in one 12-month period.
Plus at least two of the following	
Recurrent Genital Ulceration	Aphthous ulceration or scarring, observed by a physician or patient.
Eye Lesions	Anterior uveitis, posterior uveitis, cells in vitreous on slit lamp examination, or retinal vasculitis observed by an ophthalmologist.
Skin Lesions	Erythema nodosum observed by a physician or patient, pseudofolliculitis, or papulopustular lesions; or acneiform nodules consistent with Behçet’s Disease in postadolescent patients not on corticosteroid treatment.
Positive Pathergy Test	Read by a physician at 24–48 h.

## Data Availability

No new data were created, or where data is unavailable due to privacy or ethical restrictions, a statement is still required.
